# Supplementary figures and images for: Epistatic interaction between ERAP2 and HLA modulates HIV-1 adaptation and disease outcome in an Australian population
Source: PLoS Pathog. 2024 Jul 9;20(7):e1012359. doi: 10.1371/journal.ppat.1012359 (PMC11259285; doi:10.1371/journal.ppat.1012359)

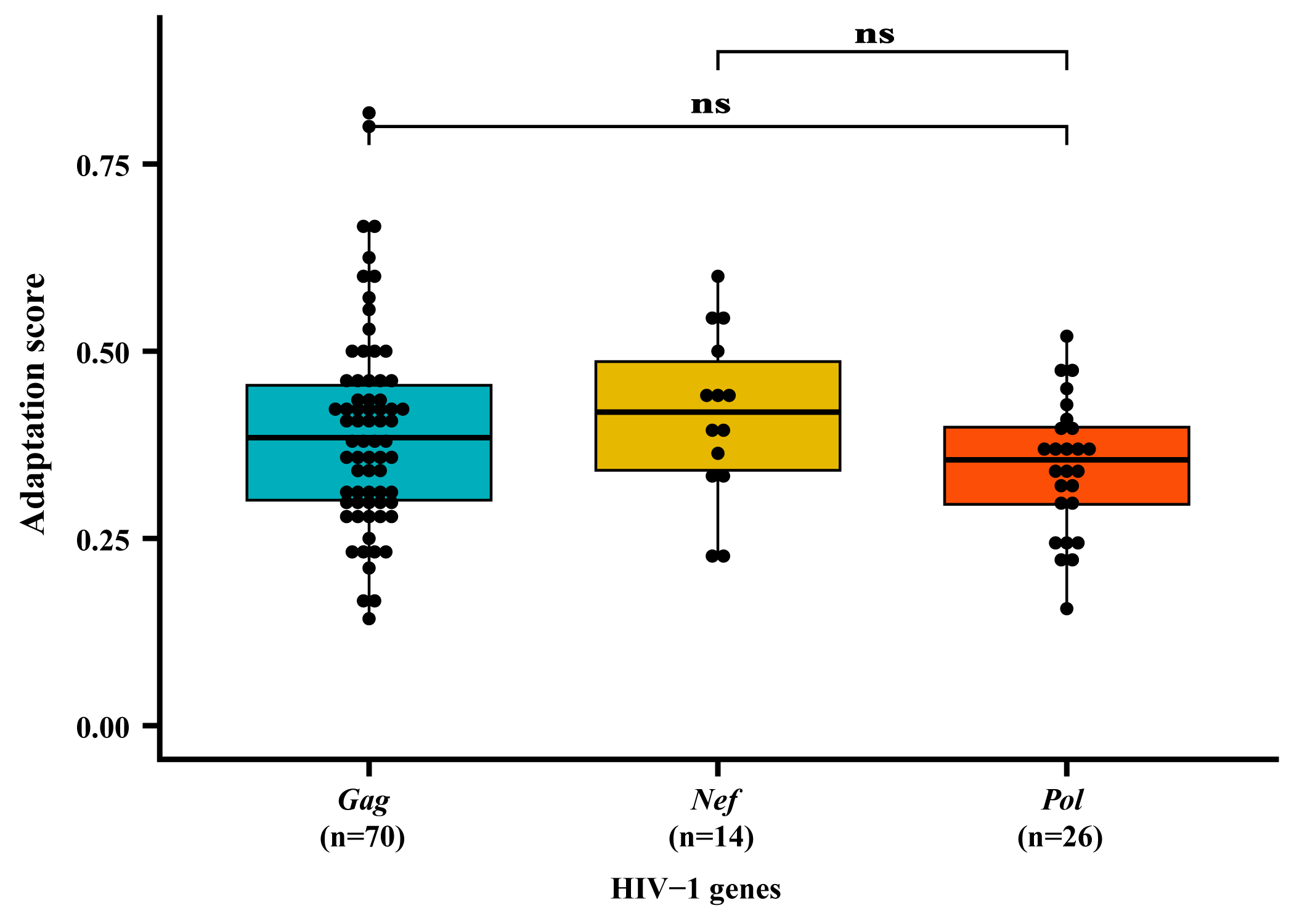

Supplement: S1 Fig — The adaptation list used in this calculation is based on statistical association of specific adaptation sites to HLA-restricted immune response described by [31]. Viral sequences with complete coverage for the adaptation sites were used in this analysis. Kruskal-Wallis test was performed to examine the difference between viral genes and the level of the adaptations to HLA class I-restricted immune responses. (TIF) [file ppat.1012359.s012.tif]

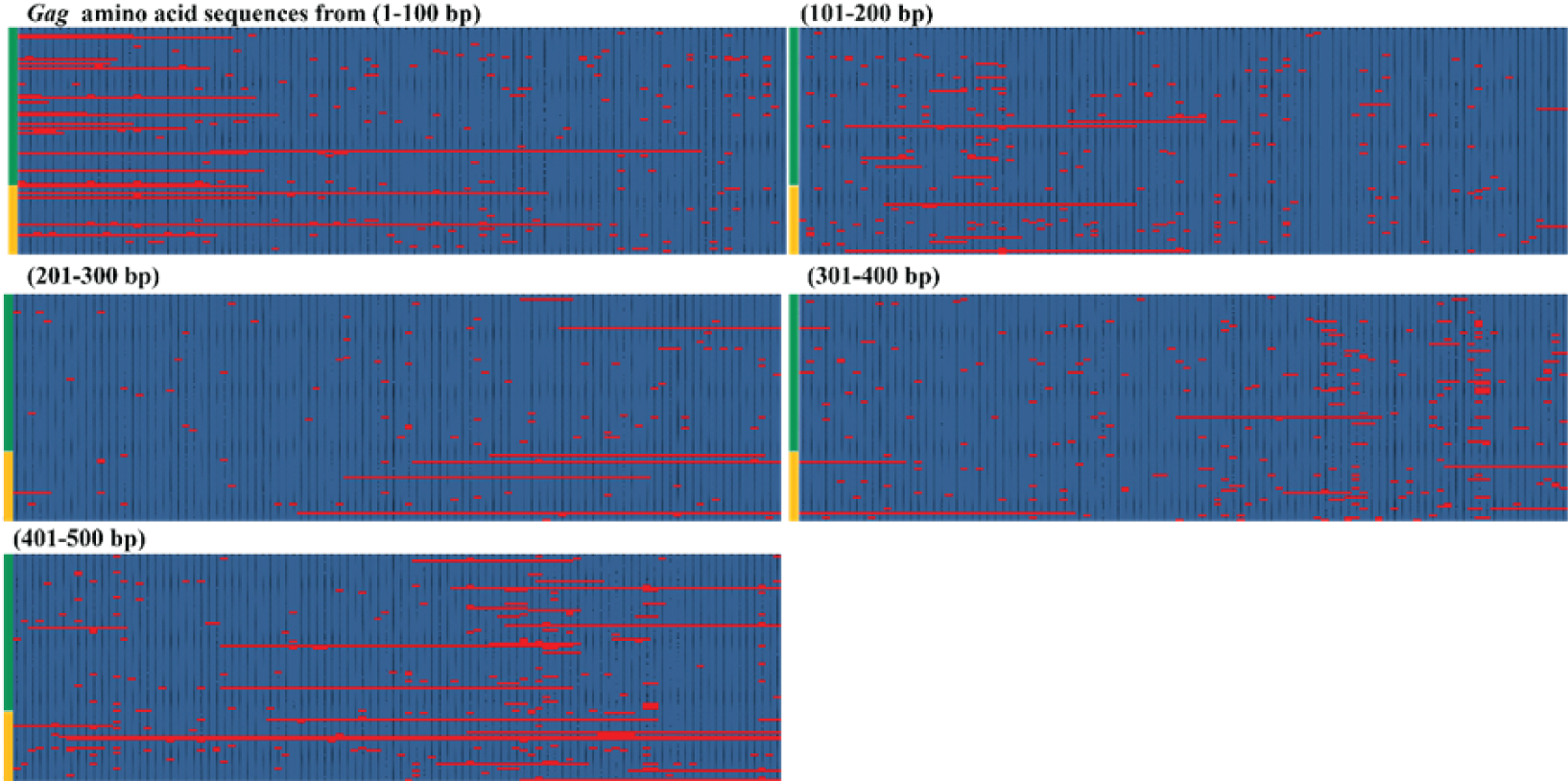

Supplement: S2 Fig — This map represents sequence coverage for 70 individuals (highlighted with the green side ribbon) with complete coverage of the possible adaptation sites, and additional 31 (highlighted with the yellow side ribbon) that have between 90 to 96% of sequence coverage at the adaptation sites. Missing sequences in the map colored in red, whereas blue represents the presence of sequence. (TIF) [file ppat.1012359.s013.tif]

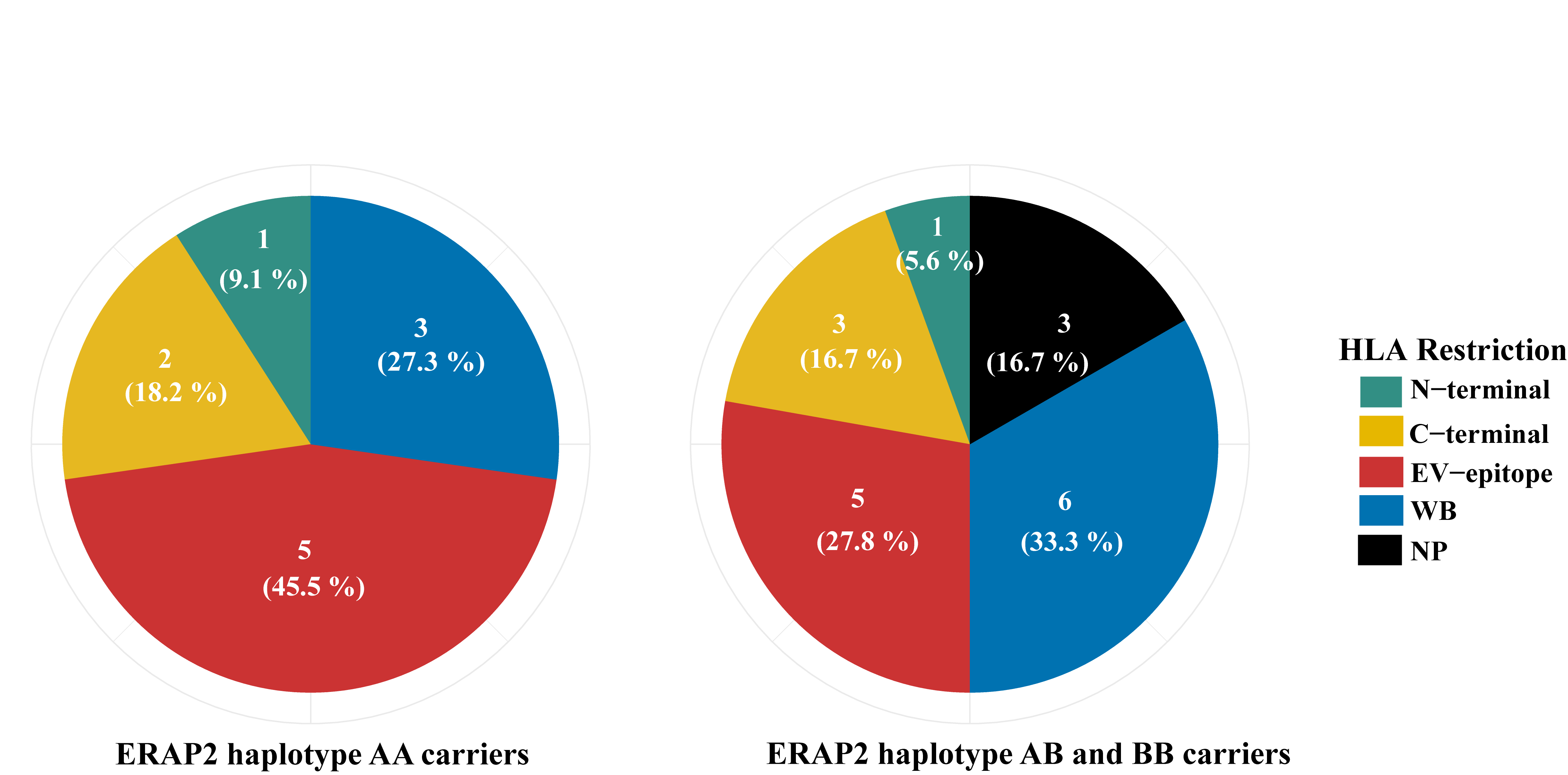

Supplement: S3 Fig — A threshold of <0.01 p-value was applied to filter the top significant associations in both groups. EV = experimentally verified. NP = not predicted. WB = weak binder. N-terminal and C-terminal indicates sites within 10 amino acids flanking epitopes. (TIF) [file ppat.1012359.s014.tif]
