# Supplementary material for: Epistatic interaction between ERAP2 and HLA modulates HIV-1 adaptation and disease outcome in an Australian population
Source: PLoS Pathog. 2024 Jul 9;20(7):e1012359. doi: 10.1371/journal.ppat.1012359 (PMC11259285; doi:10.1371/journal.ppat.1012359)
Supplement: Attachment — Submitted filename: Rebuttal letter_Al-kaabi et al 2024.pdf [file ppat.1012359.s017.pdf]

THE UNIVERSITY OF WESTERN AUSTRALIA
Achieve International Excellence

Associate Professor Silvana Gaudieri
School of Human Sciences
University of Western Australia
silvana.gaudieri@uwa.edu.au

5th January 2024

Editors-In-Chief

PLOS Pathogens

Re: Al-kaabi et al. paper no PPATHOGENS-D-23-01778

Dear Editors,

Thank you for the opportunity to provide a revised version of our manuscript PPATHOGENS-D-23-01778 **"Epistatic interaction between ERAP2 and HLA modulates viral adaptation and HIV-1 disease outcome"** for consideration for publication in your journal.

Please find attached our revised paper, with the point-by-point responses to the reviewers' comments described below. We hope that you find the responses to the reviewers' comments addresses their concerns and is now suitable for publication in *PLOS Pathogens*.

Please note that we have now removed Mr Shay Leary from the author listing following his request (communication with journal on 21st October 2023). We are now in the process of acquiring documentation of consent from all co-authors.

Regards

Silvana Gaudieri

Responses to the reviewers' comments on paper PPATHOGENS-D-23-01778

<u>**Reviewer #1**</u>

Major Comments

1.1 Although the premise of this study is interesting, this reviewer's concern is that they are based on a relatively small sample size and not validated in additional studies, thereby contributing to the large literature on HIV host genetics that remain a single finding without additional validation. Can the authors address this concern and validate in additional samples? There are many previously published studies with genome-wide data, HLA types and viral sequence data which can be mined for replication (eg. Brumme Lab, Carrington Lab etc).

Response:

We agree that replication of our findings in more samples or in an independent dataset would be ideal. However, we have utilized a unique retrospective cohort in this study, recruited prior to 2002. Since around 2015, rapid initiation of ART therapy either immediately (same day) or soon after a diagnosis in a "test and treat' environment has become standard of care in Australian, European and US guidelines. This has limited enrollment of individuals in the required numbers to natural history studies of host or viral determinants of HIV outcomes. To replicate our analyses, we require access to linked genome wide SNP typing, high resolution HLA genotyping, near full length HIV sequencing and viral load/CD4 T cell data prior to ART.

To directly address the reviewer's comment, we reviewed five genome wide association studies (GWAS) in the literature that have examined the impact of host genetic factors on HIV infection outcome in detail (Bartha et al., 2013; Fellay et al., 2009; Fellay et al., 2007; McLaren et al., 2015; Pereyra et al., 2010) (see Table 1 for more information on each of the studies). None of the studies provide the linked clinical, host and viral genotyping raw data necessary to examine the interaction between ERAP2 SNPs and HLA-restricted HIV adaptation explaining the variance of viral load across a population, which is the main finding from our study.

The study by Felley et al. (2007) provided a link to all the raw p values of individual SNP associations with set-point viral load (spVL). The SNP rs2549782, which is one of the two significant ERAP2 SNPs in our study and in complete linkage disequilibrium with rs2248374 tagging the ERAP2 haplotype B, was not significantly associated with spVL (p=0.6427). SNPs associated with ERAP2 did not appear in the top 500 significant SNPs shown in the Fellay et al. (2009) study or within the top 300 significant SNPs reported by Pereyra et al. (2010). This is not unexpected given the findings of our own analyses found that the association of the ERAP2 SNPs with viral load alone (not accounting for HLA-viral polymorphism associations) had a moderate effect size. Such associations may not reach the stringent GWAS significance threshold, as reviewed in (Tam et al., 2019). Larger sample sizes are likely required for the detection of associations outside of the two regions (major histocompatibility complex including the HLA loci and CCR5) that contain the well-established host genetic factors associated with HIV disease outcomes (Tough & McLaren, 2019), but as indicated by ourselves and the authors of the largest GWAS study in Table 1 (McLaren et al., 2015), it is unrealistic to increase sample size in studies of HIV infection outcome due to the rapid initiation of ART therapy. As such, these authors suggested that all prior GWAS on HIV infection outcome were underpowered to detect variants with modest effect size (McLaren et al., 2015).

In addition, genes subject to transcriptional regulation, alternate splicing, epistasis and epigenetic influences are less likely to be revealed by the GWAS approach. ERAP2 expression as alternate isoforms is strongly influenced by pro-inflammatory environments and within certain cell types. A recent pre-print study shows that the modulation of ERAP2 function, marked by the rs2248374 SNP,

resulted in both quantitative and qualitative changes in the cancer immunopeptidome. Studies continue to highlight differential roles of ERAP1 and 2 in shaping immunopeptidomes in keeping with the known interactions between ERAP and HLA molecules (Clark et al., 2023).

We feel that the associations in our unique dataset are robust, in keeping with known biological interactions between ERAP and HLA and we hope that further studies in other populations will be made possible by publication of our findings. We have included our analysis of other GWAS in the discussion as well as their limitations in examining the interaction and the need for further studies to assess the generalizability of our findings in other settings (lines 358-371).

Table 1: Overview of GWAS examining role of host genetic factors on HIV infection outcome

GWAS Study title (Authors)	*Number of subjects (ethnicity)*	*Disease variable*	*Data reported/availability*[*]	*Chip used*[#]
A Whole-Genome Association Study of Major Determinants for Host Control of HIV-1 (Fellay et al., 2007)	486 (Caucasian)	spVL and longitudinal VL	All raw p-values generated from GWAS	Illumina HumanHap550
Common genetic variation and the control of HIV-1 in humans (Fellay et al., 2009)	2362 (Caucasian)	spVL	Top 500 significant SNPs associated with spVL and the top 500 significant SNPs associated with disease progression	Illumina HumanHap550<br>Illumina Human1M<br>HLA chip: customized MHC-chip containing 8000 SNPs
The major genetic determinants of HIV-1 control affect HLA class I peptide presentation (Pereyra et al., 2010)	3,622 (multiple)	spVL	Only the top 300 significant SNPs in the HLA region	Illumina650kY<br>Human-1M-duo
A genome-to-genome analysis of associations between human genetic variation, HIV-1 sequence diversity, and viral control (Bartha et al., 2013)	698 (Caucasian)	VL	Significant associations of HIV genome with human genome but not with VL	Affymetrix6<br>Illumina1M<br>IlluminaOmniExpress12v1H<br>Illumina650k<br>Illumina HumanHap550<br>Illumina660W-Quad
Polymorphisms of large effect explain the majority of the host genetic contribution to variation of HIV-1 virus load (McLaren et al., 2015)	6,315 (Caucasian)	spVL	Only significant p values in the HLA region	Illumina650k<br>Affymetrix 6.0<br>Illumina 300<br>Illumina 1M<br>Illumina HumanHap550

[*]No studies had published individual linked data that could be utilized for replication of results in our study.
[#]Illumina SNP chips used in these studies contained the ERAP2 rs2549782 SNP relevant in our study but not rs2248374. However, these SNPs are in strong LD with r^2= 1 in the 1000 Genome Project.

Minor Comments

1.1 *Table -1 – Important parameters and the N and % are listed. Column 3 header should be changed (Number (%)) may not be the correct heading for Log viral load and CD4+ T cell count etc.*

Response: Table 1 column indicating "Number (%)" now also includes column header "Median (IQR)" to account for all data types.

1.2 *Change-Results line 130-(Fig-1 (A?)*

Response: The r^2 values are in Fig 1A and the D` values are in Fig 1B. We have changed the text to be "Fig 1A and B" to reflect order shown.

1.3 *Change -Results line 155- (Fig -1 (B?)*

Response: The r^2 values are in Fig1A and the D` values are in Fig 1B. We have changed the text to be "Fig 1A and B".

1.4 *Results line # 156 to line #172 describing "ERAP2 haplotype B carrying a truncated version of ERAP2 predicts better HIV infection outcomes"-are described in Figures 3A, 3B and 3C. While Results line # 178 to Line #188 describing "viral adaptation to HLA restricted T cell immune response to Gag correlate significantly with clinical outcome ", are described in Figure 2. Suggest that Figures are numbered in the order as they are mentioned in the text.*

Response: We have renumbered the figures to align with their order of mention in the Results section.

<u>**Reviewer #2**</u>

Major Comments

1. *One concern I have is the reproducibility of the ERAP2 association. The authors cite several GWAS of HIV viral load (refs 2-4) that have larger sample sizes that the study presented here. However, to my knowledge none of those identified ERAP2 SNPs as being associated with viral load. If those data are publicly available, it would be interesting to know if the ERAP2 SNPs are even nominally associated in those studies and show similar effect directions. This would increase the confidence in the reported associations and could be done without much additional effort.*

Response: Please refer to the response provided in the "Major Comments" section of Reviewer #1.

2. *I also wonder how robust the association is to potential bias due to population stratification. The authors do state that they account for race, however, they use a binary covariate (Caucasian vs Other) to achieve this. This may not be sufficient if the other category is itself quite diverse with varying viral loads in different ancestry groups. Indeed the frequency of the ERAP2 polymorphisms can vary up to 10% across continental ancestry groups (at least in 1000Genomes data) and the haplotype frequencies presented in table S2 are quite different. One way to assess this may be to compare mean viral loads between the ancestry groups and/or to perform a subset analysis in the Caucasian only individuals.*

Response: We now have conducted additional analyses by including all ethnic groups as well as performing a subset analysis solely on Caucasians. <u>Including the race covariate with all ancestry groups showed no significant change in the P-values.</u> Sub-setting the analysis to Caucasians only (173 out of 249 subjects) showed a significant association at the nominal significant level but not after FDR multiple comparisons correction, except for the additive model that remained significant after correction. Of note, we did not originally present the results of the additive genetic model as it showed similar findings to the dominant model when using race as a binary covariate in the original analysis. Within the Caucasian group, the additive model showed stronger evidence than other genetic models with log viral load but not with $CD4^+$ T cell counts. The additive model assumes that the risk allele provides independent additive risk and offers advantages over the codominant model in cases when a linear relationship can be assumed as it has greater power to reveal differences in the response variable (González & Cáceres, 2019).

The results are presented below in Tables 2-7 (and can be included in the supplementary material if needed):

Table 2: Association of ERAP2 SNPs with log viral load (Log RNA copies/ml)

**Statistical model**	**rs2549782**						**rs2248374**					
	**Codominant**		**Dominant**		**Additive**		**Codominant**		**Dominant**		**Additive**	
	**P-value**	**P-adjusted**	**P-value**	**P-adjusted**	**P-value**	**P-adjusted**	**P-value**	**P-adjusted**	**P-value**	**P-adjusted**	**P-value**	**P-adjusted**
Race Caucasians vs other	0.0012	0.0081	0.0003	0.0024	0.0008	0.0047	0.0014	0.0081	0.0004	0.0024	0.0008	0.0047
Race with all ethnic groups	0.0017	0.0109	0.0006	0.0043	0.0006	0.0040	0.0018	0.0109	0.0007	0.0043	0.0007	0.0040
Caucasians only	0.0347	0.1390	0.0209	0.1079	0.0099	0.0363	0.0312	0.1390	0.0180	0.1079	0.0090	0.0363

Note: The FDR correction is applied to include all the tested ERAP1 and ERAP2 SNPs (12 tests for each genetic model). Results obtained by fitting ANOVA model adjusted for sex, race and CCR5 delta 32 deletion. Analysis performed using SNPassoc package in RStudio. The Race Caucasian vs other results is as presented in the manuscript and added here for comparison.

Table 3: Association of ERAP2 SNPs with $CD4^+$ T cell counts (cells/µl)

Statistical model	***rs2549782***				***rs2248374***			
	Codominant		***Dominant***		***Codominant***		***Dominant***	
	P-value	***P-adjusted***	***P-value***	***P-adjusted***	***P-value***	***P-adjusted***	***P-value***	***P-adjusted***
Race Caucasians vs other	0.0326	0.1955	0.0096	0.0576	0.0143	0.1714	0.0043	0.0511
Race with all ethnic groups	0.0398	0.1840	0.0113	0.0679	0.0174	0.1840	0.0049	0.0592
Caucasians only	0.2758	ns	0.1952	ns	0.2064	ns	0.1457	ns

Note: The FDR correction is applied to include all the tested ERAP1 and ERAP2 SNPs (12 test for each genetic model). Results obtained by fitting ANOVA model adjusted for sex, race and CCR5 delta 32 deletion. Analysis performed using SNPassoc package in RStudio. $CD4^+$ T cell counts were transformed using Box-Cox transformation (lambda=0.4627871). The Race Caucasian vs other results is as presented in the manuscript and added here for comparison. ns = not significant

Table 4: Association of ERAP2 haplotypes with log viral load and $CD4^+$T cell counts using Codominant inheritance model (AA vs AB vs BB)

Statistical model	***Association with log viral load***	***Association with $CD4^+$ T cell counts***
	P-value	***P-value***
**Race Caucasians vs other**	0.0007	0.0088
**Race with all ethnic groups**	0.0011	0.0119
**Caucasians only**	0.0312	0.1731

The Race Caucasian vs other results is as presented in the manuscript and added here for comparison.

Table 5: Pairwise comparison of the association between ERAP 2 haplotypes and log viral load (RNA copies/ml) using codominant inheritance model (AA vs AB vs BB)

	Race Caucasians vs other				*Race with all ethnic groups*				*Caucasians only*			
ERAP2 Haplotypes	***Difference in Log Viral load***	***CI (Lower limit)***	***CI (Upper limit)***	***P.adjusted^***	***Difference in Log Viral load***	***CI (Lower limit)***	***CI (Upper limit)***	***P.adjusted^***	***Difference in Log Viral load***	***CI (Lower limit)***	***CI (Upper limit)***	***P.adjusted^***
AB - AA	-0.4	-0.8	-0.1	**0.0037**	-0.4	-0.7	-0.1	**0.0111**	-0.3	-0.6	0.1	0.1727
BB - AA	-0.6	-0.9	-0.2	**0.0019**	-0.5	-0.9	-0.2	**0.0018**	-0.5	-0.9	0.0	**0.0338**
BB - AB	-0.1	-0.4	0.2	0.7232	-0.2	-0.5	0.2	0.5131	-0.2	-0.6	0.2	0.5192

^P values were adjusted using Tukey multiple comparisons of means 95% family-wise confidence level in R with the adjustment sex, race and CCR5 delta 32 deletion. The Race Caucasian vs other results is as presented in the manuscript and added here for comparison.

Table 6: Pairwise comparison of the association between ERAP 2 haplotypes and CD4+T cell counts using codominant inheritance model (AA vs AB vs BB)

	Race Caucasians vs other				*Race with all ethnic groups*				*Caucasians only*			
ERAP2 Haplotypes	***Difference in $CD4^+$ T***	***CI (Lower limit)***	***CI (Upper limit)***	***P.adjusted^***	***Difference in $CD4^+$ T***	***CI (Lower limit)***	***CI (Upper limit)***	***P.adjusted^***	***Difference in $CD4^+$ T***	***CI (Lower limit)***	***CI (Upper limit)***	***P.adjusted^***
AB - AA	6.2	1.4	11.1	0.0075	5.8	1.0	10.5	0.0122	4.1	-1.1	9.4	0.1555
BB - AA	4.9	-0.8	10.5	0.1039	4.9	-0.6	10.4	0.0930	2.3	-3.9	8.5	0.6503
BB - AB	-1.3	-6.3	3.6	0.8000	-0.9	-5.8	4.0	0.9027	-1.8	-7.5	3.8	0.7264

*$CD4^+$ T cell counts transformed using Box-Cox transformation (lambda=0.4627871). ^P values were adjusted using Tukey multiple comparisons of means 95% family-wise confidence level in R with the adjustment sex, race and CCR5 delta 32 deletion. The Race Caucasian vs other results is as presented in the manuscript and added here for comparison.

Table 7: Association of ERAP2 haplotypes with log viral load and CD4+T cell counts using dominant inheritance model (AA vs AB and BB)

Statistical model	*Association with log viral load*		*Association with CD4+ T cell counts*	
	P-value	*P-adjusted*	*P-value*	*P-adjusted*
**Race Caucasians vs other**	0.0002	0.0002	0.0025	0.0030
**Race with all ethnic groups**	0.0004	0.0006	0.0032	0.0040
**Caucasians only**	0.0180	0.0206	0.0837	0.0918

*$CD4^+$ T cell counts transformed using Box-Cox transformation (lambda=0.4627871). ^P values were adjusted using Tukey multiple comparisons of means 95% family-wise confidence level in R with the adjustment sex, race and CCR5 delta 32 deletion. The Race Caucasian vs other results is as presented in the manuscript and added here for comparison.

3. *Statistical tests and thresholds used vary throughout the manuscript without much justification as to why the tests were chosen and when/ why it is appropriate to not account for multiple comparisons. For example, when comparing HLA and viral sequence polymorphism stratified by ERAP2 haplotype, I don't understand why the Fisher's exact test was used and no multiple comparison correction was applied. The authors suggest the sample size is limited but n=68 and n=180 are roughly inline with the full cohort size. It would be interesting to know if the strongest associations (i.e. those most likely to be true) are more often found in canonical or predicted epitopes.*

Response: We corrected for multiple comparisons in all the analyses in this manuscript except for the comparison of HLA and HIV-1 polymorphism stratified by ERAP2. In this specific analysis, we tested the association of 29 HLA alleles (the most common in the cohort with an allele frequency above 5%) with 500 positions across the HIV-1 *Gag* gene for each ERAP2 haplotype (AA vs AB & BB). This resulted in approximately 4730 tests for the 180 carriers of haplotype AB & BB and around 4650 tests for the 80 carriers of haplotype AA. With this number of tests relative to the size of the cohort, no position retained significance after multiple comparison. To address this, we turned to biological relevance, evaluating whether the associated positions aligned with previously published epitopes or could be predicted using bioinformatics tools such as NetMHCpan 4.1. Furthermore, the study by Bartha et al (2013) (Table 1) identified four polymorphic sites within the HIV Gag protein associated with ERAP2 SNPs reflecting putative pressure on the virus by ERAP2 (as suggested by our own analysis).

We also applied a more stringent significance threshold of p-value < 0.01 to examine the location of the strongest associations in canonical or predicted epitopes. Figure 1 shows the distribution of the 11 HLA associations with Gag amino acid positions in the ERAP2 AA haplotype carrier group and the 18 sites for the ERAP2 AB and BB haplotype carrier group. For both groups, the majority of sites fell within predicted or known CD8+ T cell epitopes. We can include this figure in the supplementary material if needed.

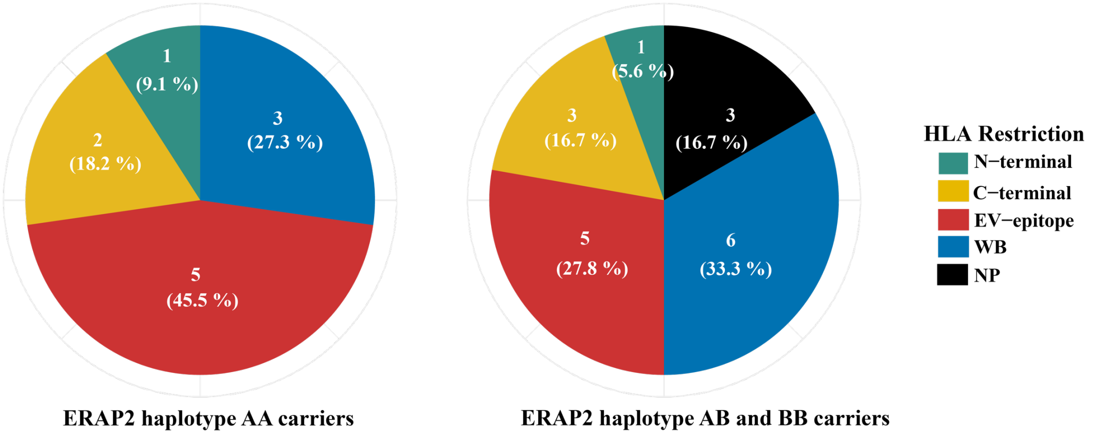

Figure 1: The distribution of the top significant associations of HLA alleles with Gag sequence among subjects carrying different ERAP2 haplotypes derived from S6 Table. A threshold of <0.01 p-value was applied to filter the top significant associations in both groups. EV = experimentally verified. NP = not predicted. WB = weak binder. N-terminal and C-terminal indicates sites within 10 amino acids flanking epitopes.

4. *The methods used to determine the amount of variability explained by the various model parameters is also not clear. In particular, when including the interaction term into the model they observe a dramatic increase in the variance explained (39%). However, it is not clear to me that this accounts for the fact that adaptation score is highly related to HLA type and therefore is independent of a large HLA effect. Some additional description of how this analysis was performed would be informative here.*

Response: We have further clarified the method used in estimating the variance in the Methods/Statistical analysis section (lines 510-515). We also added a detailed footnote to S8 Table regarding the analysis and the model used.

We applied multiple regression models to investigate the amount of variance explained by host and viral genetic factors (subjects' demographics, race, the carriage of CCR5 Δ32 deletion, carriage of known HLA alleles (HLA-B*57 and B*27) that control HIV-1 infection, adaptation score of *Gag* gene, HIV-1 clade type, carriage of different ERAP2 haplotypes). Specifically, we examined the coefficient of determination (R^2), which is the total variance explained by the factors included in each regression model, and the adjusted R^2 that shows total variance explained by the model adjusted for the number of variables used in the model.

In the first model, we focused on host genetic factors: sex, racial groups, CCR5 Δ32, HLA-B*57 and HLA-B*27 (n=249 subject). The total variance explained by all these factors measured by R^2 =17.67 %. We then fitted another regression model and incorporated all the factors in the first model in addition to the carriage of different ERAP2 haplotypes. We found that the total explained variance is 22.85 % with an increase of 5.2% attributed to variations in ERAP2 haplotypes.

In the third model, where HIV-1 clade type was introduced, there was no significant increase in variance explained (R^2=22.9%) compared to the second model. Then to examine the variance explained by the adaptation score and the interaction effect with ERAP2, we restricted the cohort to 70 subjects with 100% sequence coverage of potential adaptations in the Gag HIV-1 gene to remove any bias in the sequence coverage across the *Gag* gene and to provide equal coverage of putative adaptations for all subjects. Thus, the fourth model included all the factors tested in the first 3 models in addition to the adaptation score of *Gag* gene. We found in this subset cohort the variance explained reached 39.34 %, with an increase of 16.44 % in the variance explained than in the whole cohort. This is mainly due to incorporating the adaptation score in the model that showed a significant correlation with viral load (Spearman, p-value 0.0103) and this was done in a subset of 70 subjects. The final model was the same as the fourth model but with the addition of the interaction term between the adaptation score and the ERAP2 haplotypes. The total variance explained in the last model reached 45.08 % with 5.74 % of the variance attributed to the interaction effect. This finding shows that both viral and host genetic factors and their interaction should be considered to understand HIV-1 disease outcome.

Regarding the reviewer's point "*However, it is not clear to me that this accounts for the fact that adaptation score is highly related to HLA type and therefore is independent of a large HLA effect.",* we would like to provide further clarification of our analysis. In all tested models we used only the carriage of the HLA alleles HLA-B*57 and -B*27 that are well-known host factors that influence HIV-1 infection outcome and viral load. The adaptation score is a measurement of the level of escape mutations occurring as a result of HLA-restricted immune selection pressure. While it is specific to the HLA type, it concurrently signifies the diminishing influence of HLA in controlling the infection. Therefore, in every model that we used, we are showing two different aspects, the carriage of the HLA types that can control the infection and the level of escape mutations (adaptation score) calculated for each subject based on their HLA type and their autologous viral sequence.

Minor Comments

1. *Line 126 the use of the word 'independent' caused some confusion since the SNPs are highly correlated and therefore non-independent*

Response: We have changed the word 'independent' to 'non-independent'.

2. *It feels counter-intuitive to have Figure 3 precede figure 2 in the results.*

Response: The figures have been reordered to reflect the order of mention in the manuscript.

3. *Table S2 lists amino acid alleles for rs2549782 where the text uses primarily nucleic acids. Using similar coding throughout would improve ease of interpretation*

Response: S2 Table is aligned to match the coding throughout the manuscript to enhance interpretation and maintain consistency.

References:

Bartha, I., Carlson, J. M., Brumme, C. J., McLaren, P. J., Brumme, Z. L., John, M., Haas, D. W., Martinez-Picado, J., Dalmau, J., López-Galíndez, C., Casado, C., Rauch, A., Günthard, H. F., Bernasconi, E., Vernazza, P., Klimkait, T., Yerly, S., O'Brien, S. J., Listgarten, J., . . . Fellay, J. (2013). A genome-to-genome analysis of associations between human genetic variation, HIV-1 sequence diversity, and viral control. *Elife*, *2*, e01123. https://doi.org/10.7554/eLife.01123

Clark, K., Humphrey, S., Paes, W., Dragomir, I., Anderton, K., Ternette, N., Berry, M., Sabouni, J., Garside, S., Shiers, J., Quibell, M., & Joyce, P. (2023). 140 Development of first-in-class ERAP2 inhibitors to modulate the cancer immunopeptidome. *Journal for Immunotherapy of Cancer*, *11*(Suppl 1), A160. https://doi.org/https://doi.org/10.1136/jitc-2023-SITC2023.0140

Fellay, J., Ge, D., Shianna, K. V., Colombo, S., Ledergerber, B., Cirulli, E. T., Urban, T. J., Zhang, K., Gumbs, C. E., Smith, J. P., Castagna, A., Cozzi-Lepri, A., De Luca, A., Easterbrook, P., Gunthard, H. F., Mallal, S., Mussini, C., Dalmau, J., Martinez-Picado, J., . . . Immunology, N. C. f. H. A. V. (2009). Common genetic variation and the control of HIV-1 in humans. *PLoS Genet*, *5*(12), e1000791. https://doi.org/10.1371/journal.pgen.1000791

Fellay, J., Shianna, K. V., Ge, D., Colombo, S., Ledergerber, B., Weale, M., Zhang, K., Gumbs, C., Castagna, A., Cossarizza, A., Cozzi-Lepri, A., De Luca, A., Easterbrook, P., Francioli, P., Mallal, S., Martinez-Picado, J., Miro, J. M., Obel, N., Smith, J. P., . . . Goldstein, D. B. (2007). A whole-genome association study of major determinants for host control of HIV-1. *Science*, *317*(5840), 944-947. https://doi.org/10.1126/science.1143767

González, J. R., & Cáceres, A. (2019). *Omic Association Studies with R and Bioconductor*. CRC Press LLC. http://ebookcentral.proquest.com/lib/uwa/detail.action?docID=5789241

McLaren, P. J., Coulonges, C., Bartha, I., Lenz, T. L., Deutsch, A. J., Bashirova, A., Buchbinder, S., Carrington, M. N., Cossarizza, A., Dalmau, J., De Luca, A., Goedert, J. J., Gurdasani, D., Haas, D. W., Herbeck, J. T., Johnson, E. O., Kirk, G. D., Lambotte, O., Luo, M., . . . Fellay, J. (2015). Polymorphisms of large effect explain the majority of the host genetic contribution to variation of HIV-1 virus load. *Proc Natl Acad Sci U S A*, *112*(47), 14658-14663. https://doi.org/10.1073/pnas.1514867112

Pereyra, F., Jia, X., McLaren, P. J., Telenti, A., de Bakker, P. I., Walker, B. D., Ripke, S., Brumme, C. J., Pulit, S. L., Carrington, M., Kadie, C. M., Carlson, J. M., Heckerman, D., Graham, R. R., Plenge, R. M., Deeks, S. G., Gianniny, L., Crawford, G., Sullivan, J., . . . Zhao, M. (2010). The major genetic determinants of HIV-1 control affect HLA class I peptide presentation. *Science*, *330*(6010), 1551-1557. https://doi.org/10.1126/science.1195271

Tam, V., Patel, N., Turcotte, M., Bossé, Y., Paré, G., & Meyre, D. (2019). Benefits and limitations of genome-wide association studies. *Nature Reviews Genetics*, *20*(8), 467-484. https://doi.org/10.1038/s41576-019-0127-1

Tough, R. H., & McLaren, P. J. (2019). Interaction of the Host and Viral Genome and Their Influence on HIV Disease. *Front Genet*, *9*, 720. https://doi.org/10.3389/fgene.2018.00720
