# Supplementary material for: Epistatic interaction between ERAP2 and HLA modulates HIV-1 adaptation and disease outcome in an Australian population
Source: PLoS Pathog. 2024 Jul 9;20(7):e1012359. doi: 10.1371/journal.ppat.1012359 (PMC11259285; doi:10.1371/journal.ppat.1012359)
Supplement: Attachment — Submitted filename: Rebuttal Letter_Resubmission_Al-kaabi et al_24_5_2024.pdf [file ppat.1012359.s018.pdf]

THE UNIVERSITY OF WESTERN AUSTRALIA
*Achieve International Excellence*

Associate Professor Silvana Gaudieri
School of Human Sciences
University of Western Australia
silvana.gaudieri@uwa.edu.au

25th May 2024

Editor-In-Chief

*PLOS Pathogens*

Re: Al-kaabi et al. manuscript - PPATHOGENS-D-23-01778

Dear Editors,

Thank you for the opportunity to provide a revised version of our manuscript PPATHOGENS-D-23-01778 **"Epistatic interaction between ERAP2 and HLA modulates viral adaptation and HIV-1 disease outcome"**.

We thank the reviewers for their valuable contribution in improving this paper and believe we have addressed the remaining issue involving the examination of publicly available data to attempt to replicate our findings. A detailed description of this additional analysis is included below. The result of the replication analysis was added to the results and discussion, with all changes tracked in the revised manuscript.

Given the contribution of Professor Jacques Fellay in providing HIV-1 datasets and support thought out the process of replicating the analysis, we have included him as co-authors in the manuscript. The change of authorship consent will be collected and sent to the journal as soon as possible.

Regards

Silvana Gaudieri

**Detailed responses to the reviewers' comments on paper PPATHOGENS-D-23-01778**

**Reviewer #1**

**Major Comments**

*1. My original concern for this paper remains that the findings of this study must be validated in additional cohorts for it to contribute to literature on host genetic effects with HIV disease outcomes. The authors could directly contact the last authors with the original raw data to validate their results:*

- *A recent paper by McLaren, P.J. et al. Nature 620, 1025–1030 (2023), cites access to individual-level genotyping data which is obtainable on request (jacques.fellay@epfl.ch).*
- *Other potential cohorts of European ancestry are listed in van Manen et al.: (Genome-wide association studies on HIV susceptibility, pathogenesis, and pharmacogenomics. Retrovirology 2012 9:70)*

**Response:**

Prior to detailing the results of our additional analyses, we believe it is important to note that our study replicated, with comparable effect size, the well documented impact of host genetic factors (i.e. carriage of the CCR5 delta32 deletion and the protective HLA alleles HLA-B*57 and -B*27) on viral load as described in previous genome-wide association studies (GWAS) and numerous candidate gene studies.

***Examining the impact of ERAP2 SNPs on viral load in other cohorts***

We contacted Professor Paul McLaren to request the data used in the McLaren, P.J. et al. (2023) publication. He kindly provided us with a dataset comprising genome-wide SNPs from HIV-infected subjects representing multiple cohorts of different ethnic backgrounds (10 European and 11 African/African American) and associated set-point viral load (spVL) data, including cohorts listed in Manen et al. (2012). This dataset did not include viral sequences and as such we were only able to examine the impact of ERAP2 SNPs on disease outcome (represented by spVL measurements) but not the main finding of our study of an interaction effect on viral load between ERAP2 haplotypes and HLA-associated HIV-1 polymorphisms of the Gag gene.

We received 12477 datapoints from 21 different cohorts in which spVL data was available for 10630 participants. ERAP2 rs2549782 genotyping was available in 14 of the 21 tested cohorts (Table 1). Mean log viral load (log copies/ml) in our study cohort (WAHIV cohort) is 4.69 (4.03-5.44), which is comparable to most of the mean log viral load values in the cohorts listed in Table 1.

Initially, we examined the cohorts with available ERAP2 rs2549782 genotyping (one of the two significant ERAP2 SNPs in our study and is in complete linkage disequilibrium (LD) with the tagging SNP of haplotype B; rs2248374 in our cohort) using an ANOVA analysis adjusted for sex where applicable. No significant association was found between the tested ERAP2 SNP and the spVL in the 14 tested cohorts (only seven of which included participants of European background). Of note, the EuroCHAVI cohort showed a trend (p=0.0579) for departure from Hardy-Weinberg equilibrium (HWE) for the ERAP2 rs2549782 genotypes which may reflect different selection pressures acting on the gene.

We next examined the other seven cohorts that did not have genotyping data for ERAP2 rs2549782 but have a proxy SNP(s) that we can use to impute ERAP2 rs2248374 genotype. Table 2 shows the proxy SNPs that were found in the ERAP2 region, and their LD results with the ERAP2 rs2248374 SNP. Using an ANOVA analysis adjusted for sex where applicable, we examined the association of the proxy SNPs (with complete LD or near complete LD with rs2248374) with spVL and there was no significant association.

An important consideration in these analyses is the likelihood of different ERAP2 haplotypes in the cohorts. In our study we assessed two ERAP2 SNPs in complete LD but these are unlikely to capture

the haplotype diversity that exists in the ERAP2 region for all cohorts. Although the rs2244374 SNP assessed in our study can be used to define the broad haplotypes A and B, these are further sub-divided by other SNPs in the region into haplotypes with low frequencies (Cagliani et al., 2010; Raja & Kuiper, 2023). The usefulness of proxy SNPs is particularly susceptible to haplotype diversity. For example, in the cohort of "Laboratory for Genomic Diversity" in Table 2, there were two proxy SNPs that were in complete LD with rs2248374 based on the 1000 Genome Project data but showed different minor allele frequencies, reflecting the presence of different ERAP2 haplotypes. Differences in ERAP2 haplotype structure in different groups has been shown to impact association outcomes (Vanhille et al., 2013).

A critical difference between the GWAS datasets and our own is in the viral load measurement. While in our study, the viral load measurement was the first pre-treatment viral load taken at clinical presentation, in McLaren, P.J. et al. (2023) spVL was defined as "the log10-transformed **average number** of HIV-1 RNA copies per ml of plasma from **at least two** viral load measurements during the chronic phase of infection and before the initiation of antiretroviral therapy". In most other GWAS studies using spVL, we found that spVL was defined by taking the mean of two or more measurements during the chronic stage of infection (Bartha et al., 2013; Fellay et al., 2009; Fellay et al., 2007), and in some instances over a period of three years and diverging by no more than 0.5 log (Fellay et al., 2009). These differences in how viral load is assessed may impact the ability to determine moderate to small effect sizes as for ERAP2 in our study (estimated difference in HIV-1 log viral load between carriers of ERAP2 haplotype AA vs BB did not exceed -0.6 (95% CI: -0.9, -0.2) log.

***Examining the interaction effect on viral load between ERAP2 haplotypes and HLA-associated HIV-1 adaptation score of Gag gene in another cohort***

To replicate the interaction effect on viral load between ERAP2 haplotypes and the level of Gag adaptation to HLA class I-restricted immune response, we requested a HIV-1 GWAS dataset that has viral genomic sequences from Professor Jacques Fellay (the Swiss HIV Cohort Study (SHCS) reported in (Bartha et al., 2013). He kindly provided us with this dataset, in which we obtained GWAS genotyping data including the ERAP2 rs2549782 SNP, viral sequences of Gag gene and spVL data.

The SHCS cohort includes individuals of European ancestry and infected mainly with HIV-subtype B. We received genotyping data of 3018 subjects; however, after combining the genotyping data with viral load and viral genetic sequences (Gag gene), we were left with 163 eligible participants to replicate the interaction analysis. We first performed HLA imputation (to 4-digit resolution) for the SHCS cohort using "HIBAG" package in RStudio (Zheng et al., 2014). We compared the imputation accuracy of "HIBAG" package using another dataset that has been previously imputed with the SNP2HLA package (sample size= 516) and we found that the results of the HLA imputation were highly concordant. After imputing HLA, we used the SHCS HLA-class I genotypes to calculate HLA-associated HIV-1 adaptation score of the Gag gene as reported in our manuscript. We took viral sequence coverage into account and used that as a covariate to select data with complete sequence coverage at the possible HLA-associated adaptation sites. Next, we examined the correlation between spVL and the HLA associated HIV-1 adaptation score of Gag gene using the Spearman correlation test. We did not find a significant correlation between viral adaptation in Gag and spVL (Figure 1). We next fitted an ANOVA model to investigate the interaction effect between ERAP2 rs2549782 genotype and viral adaptation score of Gag gene on spVL. There was no significant interaction effect detected in this analysis. By the loss of association signal between HLA associated HIV-1 adaptation score of Gag and spVL, we did not expect to find a significant interaction effect.

The association between overall autologous HLA-associated HIV-1 adaptation score (calculated for near full-length of HIV-1 genome) and HIV-1 clinical outcome has been well documented (Carlson et al., 2016). Th e fact that we did not find a significant association between spVL and the adaptation score in the SHCS cohort is not surprising. In Carlson et al. (2016), the authors found that the Gag-specific adaptation score was significantly associated with VL (defined as a cross-sectional single measurement viral load taken mostly upon first presentation) in the Southern Africa (HIVC) cohort (P=0.03) but not in the British Columbia (HIVB) cohort (P=0.51), although in both cohorts the overall adaptation score was significantly associated with VL ($P= 1*10^{-16}$, Southern Africa (HIVC) cohort; P=0.03, British

Columbia (HIVB) cohort; in Supplementary Table 1 in (Carlson et al., 2016)). The authors note in Supplementary Note 2 of the paper that the majority of the British cohort were individuals of advanced clinical stage and this may have impacted the relationship between adaptation and viral load.

Overall, the results in our study were not replicated in any of the cohorts previously reported in (McLaren et al., 2023) and in the SHCS cohort. The correlation between viral load and the level of viral adaption in Gag shown in our study has been previously shown (Carlson et al., 2016). The differences in the measurement of spVL between the studies is an important factor to consider along with other variations in some of the studies such as different ethnic background, likely different ERAP2 haplotypes, restriction to specific HIV-1 subtype (GWAS did not include HIV-1 subtype) and focus on specific comparisons including elite controllers (c.f. WAHIV cohort that involved prospective enrolment of HIV-1 infected individuals with broad criteria).

Given these differences, the influence of ERAPs on HIV adaptation cannot be excluded in the other cohorts, and it is hoped that publication of our findings may lead to comparable data being generated independently as well as experimental studies of ERAP-HLA-HIV interaction. Given the extensive nature of the analysis described above and the added benefit to the overall manuscript, we have added a paragraph in the results section of the revised manuscript with an explanation of the lack of concordance between our study and previous GWAS in the discussion section.

*2. In Line 62 of the manuscript- Author summary, the word "subject" should be changed to "host" or "participant "according to the NIAID HIV language guide*

**Response:** Changed throughout manuscript as suggested.

**Minor Comments**

**Reviewer #1**

1. *Authors should provide a data availability statement*

**Response:**

We have provided a data availability statement.

**Reviewer #2**

1. *I would be in favour of including the figures/tables provided in the response as supplementary materials.*

**Response:** We have included the results as footnotes in the relevant supplementary materials as suggested.

**Changes made to the manuscript (Line number according to the tracked changes version):**

1. During the revising process, we corrected the HLA-associated HIV-1 adaptation score of *Pol* gene as there was unintentional error in the amino acid positions of the list used in the calculation. This change did not affect the main results of the study and we made changes in lines 202-210 and on S Fig 1 according to this correction.
2. We added the results of the replication analysis in the manuscript on lines 299-326; in the discussion 400-444; and provided S10 and S11 Tables in the supplementary materials.
3. Changing the word "subject" to "participant" or "host" as suggested by the reviewer throughout the manuscript.
4. Changing the word "clade" to "subtype" throughout the manuscript.

**Table 1: Description of HIV-1 GWAS dataset reported in (McLaren et al., 2023)**

| *Studies* | *Ancestry* | *Cohort size** | *Mean spVL* | *ERAP2 genotyping of rs2549782* | | | *HWE P-value (rs2549782)* | *Cohort description* | *Reference* |
|---|---|---|---|---|---|---|---|---|---|
| | | | | *TT* | *TG* | *GG* | | | |
| Amsterdam cohort study | European | 384 | 4.2 | 118 | 174 | 92 | 0.0816 | MSM & DU; Progressor & LTNP; 1984-1986 | (van Manen et al., 2011) |
| International HIV Controllers Study/AIDS Clinical Trials Group | European | 503 | 4 | NA | NA | NA | | Elite controller (n=184) & Progressor (n=321) | Pereyra et al., 2010) |
| International HIV Controllers Study/AIDS Clinical Trials Group | European | 577 | 4.1 | 145 | 294 | 138 | 0.6776 | Elite controller (n=195) & Progressor (n=383) | Pereyra et al., 2010) |
| International HIV Controllers Study/AIDS Clinical Trials Group | European | 576 | 4.4 | 145 | 307 | 123 | 0.1124 | Elite controller (n=97) & Progressor (n=483) | Pereyra et al., 2010) |
| EuroCHAVI | European | 1304 | 3.9 | 363 | 680 | 257 | 0.0579 | Participants are combined form multiple cohorts | (Fellay et al., 2009) |
| GRIV/PRIMO | European | 960 | 5 | NA | NA | NA | | | (Dalmasso et al., 2008) |
| Laboratory for Genomic Diversity | European | 1034 | 4.4 | NA | NA | NA | | 97% Male; combined from multiple cohorts | (Hendrickson et al., 2010) |
| Multicentre AIDS Cohort Study | European | 752 | 4.3 | 184 | 391 | 171 | 0.2129 | | (Herbeck et al., 2010) |
| University Health Study | European | 239 | 4.5 | 71 | 124 | 44 | 0.5122 | | (Johnson et al., 2015) |
| Multicentre AIDS Cohort Study | European | 365 | 4.2 | 95 | 182 | 88 | 1.0000 | | (Herbeck et al., 2010) |
| International HIV Controllers Study/AIDS Clinical Trials Group | African American | 412 | 4.3 | 140 | 194 | 77 | 0.5442 | Elite controller & Progressor | Pereyra et al., 2010) |
| International HIV Controllers Study/AIDS Clinical Trials Group | African American | 378 | 4.2 | 119 | 190 | 69 | 0.6767 | Elite controller & Progressor | Pereyra et al., 2010) |
| International HIV Controllers Study/AIDS Clinical Trials Group | African American | 379 | 4 | 113 | 191 | 71 | 0.6011 | Elite controller & Progressor | (Pereyra et al., 2010) |
| International AIDS Vaccine Initiative | African | 527 | 4.5 | 115 | 253 | 125 | 0.5892 | | (Li et al., 2014) |
| International AIDS Vaccine Initiative | African | 242 | 4.4 | 65 | 115 | 62 | 0.4422 | | (Li et al., 2014) |
| Laboratory for Genomic Diversity | African American | 322 | 4.2 | NA | NA | NA | | | (Hendrickson et al., 2010) |
| Multicentre AIDS Cohort Study | African American | 515 | 4.1 | 167 | 255 | 92 | 0.7873 | | (Pelak et al., 2010) |
| Majengo Observational Cohort Study | African | 147 | 3.8 | NA | NA | NA | | Female only | (Luo et al., 2012) |
| Rural Clinical Cohort Uganda | African | 144 | 4.3 | NA | NA | NA | | | (Ssemwanga et al., 2011) |
| Swiss HIV Cohort Study | African | 340 | 4 | NA | NA | NA | | | (McLaren et al., 2023) |
| University Health Study | African American | 530 | 4.2 | 181 | 260 | 89 | 0.8578 | | (Johnson et al., 2015) |
| Total | | | 10630 | | | | | | |

*** Cohort size represents the number of participants who had spVL.**

**Table 2: ERAP2 Proxy SNPs with near complete LD with rs224837**

| *SNP rs number* | *Ancestry group* | *Cohort* | *Position on chromosome 5* | *Allele 1* | *Allele 2* | *D prime* | $r^2$ | *P-value* | *Minor allele frequency* | *HWE test (P-value)* |
|---|---|---|---|---|---|---|---|---|---|---|
| rs2161657 | European | International HIV Controllers Study/AIDS Clinical Trials Group | 96232806 | C | T | 1 | 0.996 | 0.6225 | 0.47 | 0.1068 |
| rs2548533* | European | Laboratory for Genomic Diversity | 96238401 | A | G | 1 | 1 | 0.5062 | 0.48 | 0.4934 |
| rs2549797* | European | Laboratory for Genomic Diversity | 96245518 | A | G | 1 | 1 | 0.8352 | 0.07 | 0.7993 |
| rs2548533 | African | Laboratory for Genomic Diversity | 96238401 | A | G | 0.997 | 0.991 | 0.5062 | 0.45 | 0.3100 |
| rs2549797 | African | Laboratory for Genomic Diversity | 96245518 | A | G | 1 | 0.997 | 0.8352 | 0.34 | 0.9018 |
| rs2549797 | African | Majengo Observational Cohort Study | 96245518 | A | G | 1 | 0.997 | 0.2041 | 0.42 | 0.6112 |
| rs2042383 | African | Rural Clinical Cohort Uganda | 96234621 | C | T | 1 | 1.000 | 0.1181 | 0.44 | 0.2807 |
| rs2548538 | African | Swiss HIV Cohort Study | 96232142 | A | T | 1 | 1.000 | 0.2325 | 0.50 | 0.3868 |

**Note:** Genomic positions are based on GRCH37 reference genome. The GRIV and PRIMO cohort study did not have any proxy SNPs. Results of LD are based on 1000 Genomes Project (2015) using LDlinkR R (Myers et al., 2020). * The proxy SNPs (rs2548533 and rs2549797) are in complete LD with rs2248374 but showing different minor allele frequency in the European cohort that demonstrates different ERAP2 haplotypes.

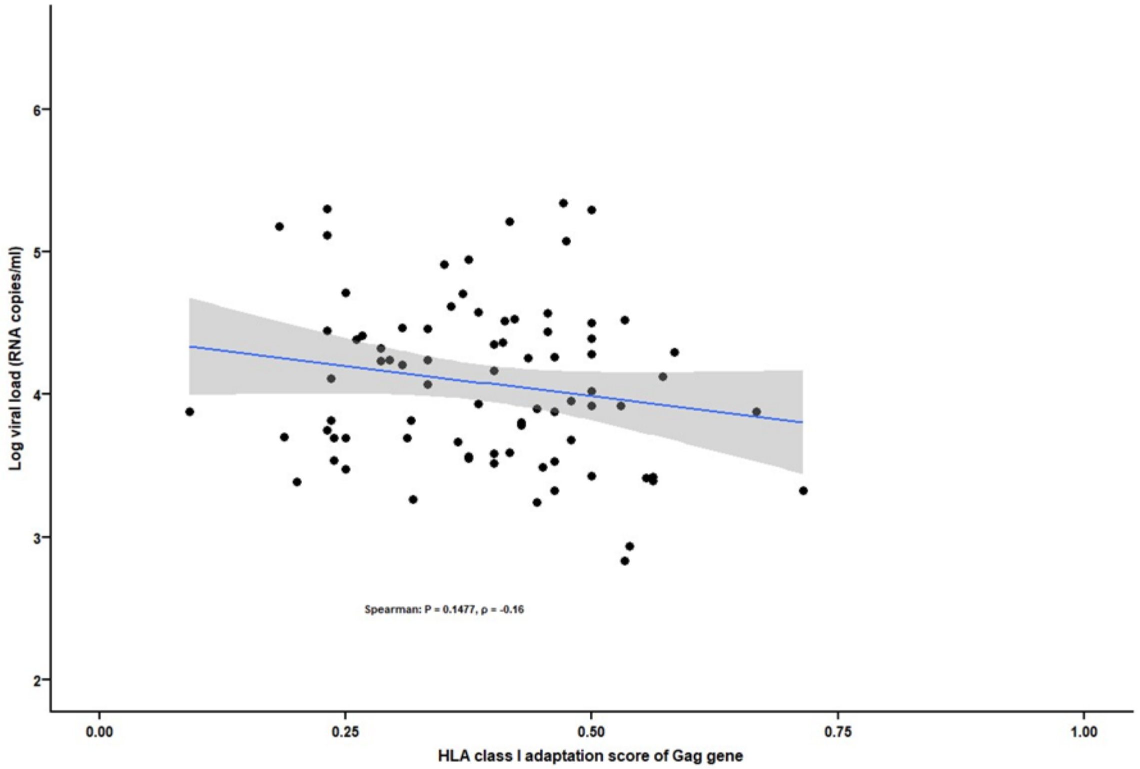


**Figure 1: Correlation of HLA-associated HIV-1 adaptation score of Gag gene with spVL in SHCS cohort**. The Spearman correlation test was used in this analysis. Only HIV-1 subtype B was included in this analysis.

**References:**


Bartha, I., Carlson, J. M., Brumme, C. J., McLaren, P. J., Brumme, Z. L., John, M., Haas, D. W., Martinez-Picado, J., Dalmau, J., López-Galíndez, C., Casado, C., Rauch, A., Günthard, H. F., Bernasconi, E., Vernazza, P., Klimkait, T., Yerly, S., O'Brien, S. J., Listgarten, J., . . . Fellay, J. (2013). A genome-to-genome analysis of associations between human genetic variation, HIV-1 sequence diversity, and viral control. *Elife*, *2*, e01123. https://doi.org/10.7554/eLife.01123

Cagliani, R., Riva, S., Biasin, M., Fumagalli, M., Pozzoli, U., Lo Caputo, S., Mazzotta, F., Piacentini, L., Bresolin, N., Clerici, M., & Sironi, M. (2010). Genetic diversity at endoplasmic reticulum aminopeptidases is maintained by balancing selection and is associated with natural resistance to HIV-1 infection. *Hum Mol Genet*, *19*(23), 4705-4714. https://doi.org/10.1093/hmg/ddq401

Carlson, J. M., Du, V. Y., Pfeifer, N., Bansal, A., Tan, V. Y., Power, K., Brumme, C. J., Kreimer, A., DeZiel, C. E., Fusi, N., Schaefer, M., Brockman, M. A., Gilmour, J., Price, M. A., Kilembe, W., Haubrich, R., John, M., Mallal, S., Shapiro, R., . . . Goepfert, P. A. (2016). Impact of pre-adapted HIV transmission. *Nat Med*, *22*(6), 606-613. https://doi.org/10.1038/nm.4100

Fellay, J., Ge, D., Shianna, K. V., Colombo, S., Ledergerber, B., Cirulli, E. T., Urban, T. J., Zhang, K., Gumbs, C. E., Smith, J. P., Castagna, A., Cozzi-Lepri, A., De Luca, A., Easterbrook, P., Gunthard, H. F., Mallal, S., Mussini, C., Dalmau, J., Martinez-Picado, J., . . . Immunology, N. C. f. H. A. V. (2009). Common genetic variation and the control of HIV-1 in humans. *PLoS Genet*, *5*(12), e1000791. https://doi.org/10.1371/journal.pgen.1000791

Fellay, J., Shianna, K. V., Ge, D., Colombo, S., Ledergerber, B., Weale, M., Zhang, K., Gumbs, C., Castagna, A., Cossarizza, A., Cozzi-Lepri, A., De Luca, A., Easterbrook, P., Francioli, P., Mallal, S., Martinez-Picado, J., Miro, J. M., Obel, N., Smith, J. P., . . . Goldstein, D. B. (2007). A whole-genome association study of major determinants for host control of HIV-1. *Science*, *317*(5840), 944-947. https://doi.org/10.1126/science.1143767

McLaren, P. J., Porreca, I., Iaconis, G., Mok, H. P., Mukhopadhyay, S., Karakoc, E., Cristinelli, S., Pomilla, C., Bartha, I., Thorball, C. W., Tough, R. H., Angelino, P., Kiar, C. S., Carstensen, T., Fatumo, S., Porter, T., Jarvis, I., Skarnes, W. C., Bassett, A., . . . Fellay, J. (2023). Africa-specific human genetic variation near CHD1L associates with HIV-1 load. *Nature*, *620*(7976), 1025-1030. https://doi.org/10.1038/s41586-023-06370-4

Myers, T. A., Chanock, S. J., & Machiela, M. J. (2020). LDlinkR: An R Package for Rapidly Calculating Linkage Disequilibrium Statistics in Diverse Populations. *Front Genet*, *11*, 157. https://doi.org/10.3389/fgene.2020.00157

Raja, A., & Kuiper, J. J. W. (2023). Evolutionary immuno-genetics of endoplasmic reticulum aminopeptidase II (ERAP2). *Genes & Immunity*. https://doi.org/10.1038/s41435-023-00225-8

Vanhille, D. L., Hill, L. D., Hilliard, D. D., Lee, E. D., Teves, M. E., Srinivas, S., Kusanovic, J. P., Gomez, R., Stratikos, E., Elovitz, M. A., Romero, R., & Strauss, J. F., 3rd. (2013). A Novel ERAP2 Haplotype Structure in a Chilean Population: Implications for ERAP2 Protein Expression and Preeclampsia Risk. *Molecular genetics & genomic medicine*, *1*(2), 98-107. https://doi.org/10.1002/mgg3.13

Zheng, X., Shen, J., Cox, C., Wakefield, J. C., Ehm, M. G., Nelson, M. R., & Weir, B. S. (2014). HIBAG--HLA genotype imputation with attribute bagging. *Pharmacogenomics J*, *14*(2), 192-200. https://doi.org/10.1038/tpj.2013.18
