# Supplementary material for: Epistatic interaction between ERAP2 and HLA modulates HIV-1 adaptation and disease outcome in an Australian population
Source: PLoS Pathog. 2024 Jul 9;20(7):e1012359. doi: 10.1371/journal.ppat.1012359 (PMC11259285; doi:10.1371/journal.ppat.1012359)
Supplement: Attachment — Submitted filename: Rebuttal Letter_Resubmission_17_6_2024.pdf [file ppat.1012359.s019.pdf]

THE UNIVERSITY OF WESTERN AUSTRALIA
Achieve International Excellence

Associate Professor Silvana Gaudieri
School of Human Sciences
University of Western Australia
silvana.gaudieri@uwa.edu.au

17th June 2024

Editor-In-Chief

PLOS Pathogens

Re: Al-kaabi et al. manuscript - PPATHOGENS-D-23-01778

Dear Editors,

Thank you for the opportunity to provide a revised version of our manuscript PPATHOGENS-D-23-01778 **"Epistatic interaction between ERAP2 and HLA modulates viral adaptation and HIV-1 disease outcome"**.

We believe the changes we have made to the revised manuscript addresses the remaining reviewer's issues. A detailed description of our response to the reviewer and changes in the manuscript are included below.

Regards

Silvana Gaudieri

Detailed responses to the reviewer's comments on paper PPATHOGENS-D-23-01778

Reviewer #1

Major Comments

1. The authors have reached out to new authors to include analyses on previously published GWAS data with ERAP2 SNPS. The ERAP2 findings unfortunately did not validate in additional cohorts. Based on their additional analyses their original findings are not substantiated even with the limitations mentioned. The authors should tone down the title, abstract and the rest of the paper substantially to reflect these updates. Include clearly in the abstract that these findings were only seen in one cohort and not validated further.

Response:

We have modified the title, abstract (lines 59-69), and discussion (lines 418-419, 433-441) to reflect the reviewer's comment.

We conclude from the results of our study and the replication analysis that the impact of ERAP2 on immune-related disease outcomes should be investigated in the context of HLA types, ERAP1 and other molecules that are involved in the immune response. Underpinning these interactions are viral adaptations that cause the loss of immune responses that also impact outcome. Importantly, GWAS will no longer provide insights into the genetic factors involved in HIV-1 control (as indicated in (McLaren et al., 2015)); investigating the epistatic interaction of viral and immune genetic factors involved in HIV-1 pathogenesis will reveal more significant associations that can inform future HIV research.

Studies on other diseases support the importance of considering these epistatic interactions in better understanding disease gene associations. In Ankylosing Spondylitis and birdshot chorioretinopathy disease, the impact of ERAP2 on the affinity of the peptidome is dependent on the risk HLA allele (Lorente et al., 2019; Martín-Esteban et al., 2017; Sanz-Bravo et al., 2018). Furthermore, ERAP1 has a range of enzymatic activities that are likely affected by physical interactions with ERAP2, impacting the peptide repertoire and T cell reactivity to specific epitopes (Evnouchidou et al., 2014; Guasp et al., 2019; Martín-Esteban et al., 2017; Tedeschi et al., 2023). These studies highlight the importance of examining the interaction of highly variable host immune-related genetic factors in the context of disease outcome.

2. The word "subject" continues to be used in the supplementary data etc.

Response:

We thank the reviewer for identifying this oversight from our last version. We have now replaced the word "subject" with "participant" or "host" throughout the supplementary materials.

Changes made to the manuscript:

Minor changes have been made to the text to improve the readability of the manuscript.

References:

Evnouchidou, I., Weimershaus, M., Saveanu, L., & van Endert, P. (2014). ERAP1-ERAP2 dimerization increases peptide-trimming efficiency. *J Immunol*, *193*(2), 901-908. https://doi.org/10.4049/jimmunol.1302855

Guasp, P., Lorente, E., Martín-Esteban, A., Barnea, E., Romania, P., Fruci, D., Kuiper, J. W., Admon, A., & López de Castro, J. A. (2019). Redundancy and Complementarity between ERAP1 and

ERAP2 Revealed by their Effects on the Behcet's Disease-associated HLA-B*51 Peptidome. *Mol Cell Proteomics*, *18*(8), 1491-1510. https://doi.org/10.1074/mcp.RA119.001515
Lorente, E., Redondo-Antón, J., Martín-Esteban, A., Guasp, P., Barnea, E., Lauzurica, P., Admon, A., & López de Castro, J. A. (2019). Substantial Influence of ERAP2 on the HLA-B*40:02 Peptidome: Implications for HLA-B*27-Negative Ankylosing Spondylitis. *Mol Cell Proteomics*, *18*(11), 2298-2309. https://doi.org/10.1074/mcp.RA119.001710
Martín-Esteban, A., Sanz-Bravo, A., Guasp, P., Barnea, E., Admon, A., & López de Castro, J. A. (2017). Separate effects of the ankylosing spondylitis associated ERAP1 and ERAP2 aminopeptidases determine the influence of their combined phenotype on the HLA-B*27 peptidome. *J Autoimmun*, *79*, 28-38. https://doi.org/10.1016/j.jaut.2016.12.008
McLaren, P. J., Coulonges, C., Bartha, I., Lenz, T. L., Deutsch, A. J., Bashirova, A., Buchbinder, S., Carrington, M. N., Cossarizza, A., Dalmau, J., De Luca, A., Goedert, J. J., Gurdasani, D., Haas, D. W., Herbeck, J. T., Johnson, E. O., Kirk, G. D., Lambotte, O., Luo, M., . . . Fellay, J. (2015). Polymorphisms of large effect explain the majority of the host genetic contribution to variation of HIV-1 virus load. *Proc Natl Acad Sci U S A*, *112*(47), 14658-14663. https://doi.org/10.1073/pnas.1514867112
Sanz-Bravo, A., Martín-Esteban, A., Kuiper, J. J. W., García-Peydró, M., Barnea, E., Admon, A., & López de Castro, J. A. (2018). Allele-specific Alterations in the Peptidome Underlie the Joint Association of HLA-A*29:02 and Endoplasmic Reticulum Aminopeptidase 2 (ERAP2) with Birdshot Chorioretinopathy. *Mol Cell Proteomics*, *17*(8), 1564-1577. https://doi.org/10.1074/mcp.RA118.000778
Tedeschi, V., Paldino, G., Alba, J., Molteni, E., Paladini, F., Scrivo, R., Congia, M., Cauli, A., Caccavale, R., Paroli, M., Di Franco, M., Tuosto, L., Sorrentino, R., D'Abramo, M., & Fiorillo, M. T. (2023). ERAP1 and ERAP2 Haplotypes Influence Suboptimal HLA-B*27:05-Restricted Anti-Viral CD8+ T Cell Responses Cross-Reactive to Self-Epitopes. *Int J Mol Sci*, *24*(17). https://doi.org/10.3390/ijms241713335
